# Quantitative analysis of retinal layers on three-dimensional spectral-domain optical coherence tomography for pituitary adenoma

**DOI:** 10.1371/journal.pone.0179532

**Published:** 2017-06-19

**Authors:** Min Sun, Zhiqiang Zhang, Chiyuan Ma, Suihua Chen, Xinjian Chen

**Affiliations:** 1 School of Electronic and Information Engineering, Soochow University, Suzhou, Jiangsu, China; 2 Department of Electronic Engineering, Huaian Vocational College of Information Technology, Huaian, Jiangsu, China; 3 Department of Medical Imaging, Nanjing General Hospital of Nanjing Military Command, Nanjing, Jiangsu, China; 4 Department of Neurosurgery, Nanjing General Hospital of Nanjing Military Command, Nanjing, Jiangsu, China; 5 Department of Ophthalmology, Nanjing General Hospital of Nanjing Military Command, Nanjing, Jiangsu, China; Weill Cornell Medical College in Qatar, QATAR

## Abstract

**Purpose:**

To quantitatively investigate the characteristics of eyes with pituitary adenoma presented by three-dimensional (3D) spectral-domain optical coherence tomography (SD-OCT) using three common indices, including thickness, optical intensity ratio, and optical intensity attenuation coefficient (OIAC).

**Methods:**

The SD-OCT database of 38 patients with pituitary adenoma and 39 normal controls were included in the study. Quadrantal and average measurements of thickness, optical intensity ratio, and OIAC were calculated for macular retinal nerve fiber layer (mRNFL), ganglion cell layer (GCL) combined with inner plexiform layer (IPL) (GCIPL) and/or the collective ganglion cell complex (GCC). The parameters of patients and controls were compared by unpaired *t*-test and Mann-Whitney *U*-test. The relationships between the optical intensity ratio and the thickness of mRNFL and GCIPL were evaluated by Pearson’s correlation. Diagnostic performances of these indices were assessed using receiver operating characteristic (ROC) analysis.

**Results:**

Significant decreases in thickness existed in the mRNFL and nasal GCC of patients compared with controls (p-values of 0.000 to 0.039). Optical intensity ratios in the relevant retinal layers of patients were almost all lower than those of controls. In patients, optical intensities were increased in the mRNFL but decreased in the GCIPL along with an increase of retinal thicknesses. The OIAC measurements were significantly higher in the upper quadrants and global average of the mRNFL in patients. The areas under the ROC curves (AUC) obtained by global average mRNFL thickness was significantly greater than that of the global average OIAC in the mRNFL (p = 0.0265).

**Conclusions:**

Thicknesses of the mRNFL and nasal GCC were significantly decreased in the retinas of patients with pituitary adenoma compared with controls. The differences of the optical intensity ratio and OIAC between patients and controls were not all statistically significant. Thickness was more sensitive than optical characteristics indices in distinguishing pituitary adenoma from controls.

## Introduction

Visual dysfunction is one of the most common symptoms of pituitary adenoma, which is caused by direct compression to the optic chiasm or disturbances in the optic chiasm’s blood supply system [1–4]. With our growing understanding of the close relationships among changes in the retina, the deterioration of visual function, and damage to the optic chiasm, the retina has gradually become the focus of research in the visual recovery of patients suffering pituitary adenoma. Optical coherence tomography (OCT), which provides noninvasive cross-sectional imaging of the retinal layers [5], has been used to evaluate morphological changes in the retina induced by pituitary adenoma and to explore the relationship between the thickness of retinal layers and the visual functions of patients [6–9].

**Fig 1:**
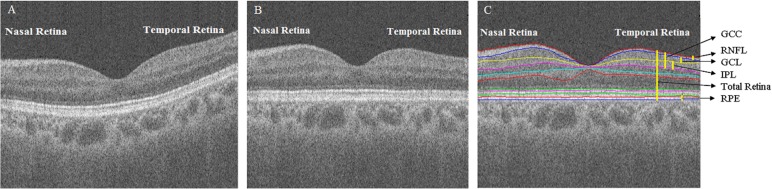
A macular SD-OCT b-scan of a healthy control. (A) Original SD-OCT b-scan image. (B) Flattened image of the original SD-OCT b-scan image. (C) Layer segmentation on the flattened image.

In addition to the morphological characteristics, the information obtained from OCT also includes the light reflectivity profile presented as image intensities, which depend on differences in the optical backscattering properties of retinal tissue [10–12]. Fig 1 shows a macular spectral-domain OCT (SD-OCT) b-scan of a healthy control, demonstrating differences in the morphology and optical intensity between retinal layers. The light reflectivity of the retinal layer will be different when pathological changes exist, even without thickness changes [10, 13–15]. To the best of our knowledge, no study in the current literature has explored the optical characteristics of pituitary adenoma retina presented by OCT, even though the existence of pathological damage to retinal axons or ganglion cells due to this kind of tumor is well known.

Optical intensity ratio and optical intensity attenuation coefficient (OIAC) are two common indices that describe the optical characteristics presented by OCT data. Direct analysis of OCT image intensity is considered to be inappropriate, because the scan data are exported from the OCT machine as unprocessed raw data and noise may be introduced due to the image quality. In the calculation of these two indices, a certain retinal layer that is assumed to have constant backscatter properties is used as a reference layer for normalization. The term attenuation coefficient was first proposed and proofed in a retinal nerve fiber layer (RNFL) study of glaucomatous eyes [16]. The OIAC is defined by layer thickness and the optical intensity ratio, combing the morphological and optical characteristic information. It is considered to be more comprehensive than either thickness or optical intensity ratio alone, and can achieve reliable measurements of changes involved in pathological processes [14, 16].

To quantitatively analyze the characteristics of pituitary adenoma retinas, the thickness, optical intensity ratio, and OIAC changes in the macular RNFL (mRNFL); the ganglion cell layer (GCL) combined with the inner plexiform layer (IPL) (GCIPL); and/or the ganglion cell complex (GCC) were analyzed using three-dimensional (3D) SD-OCT data. Correlation analyses were conducted to reveal the relationships between light reflectivity and thickness of the mRNFL and GCIPL for patients and controls. Comparisons between the patients with pituitary adenoma and normal controls were also conducted to determine whether the OCT parameters mentioned above were sensitive in the detection of retinal damage due to pituitary adenoma.

## Materials and methods

### Subjects

The SD-OCT database of 38 patients (21 male, 17 female) with diagnoses of pituitary adenoma and 39 normal controls (19 male, 20 female) were collected between August 2015 and July 2016 at the Nanjing General Hospital of Nanjing Military Command. The main patient exclusion criteria were as follows: 1) presence of congenital eye disorders, 2) a history of ocular surgery and 3) presence of any optic disc anomaly or macular disease. Healthy people with a normal ophthalmic examination were selected as the control group. This study was approved by the Institutional Review Board of the Nanjing General Hospital of Nanjing Military Command and adhered to the tenets of the Declaration of Helsinki. Informed consent was required from all subjects with verbal permission during the process of OCT inspection.

Subjects underwent SD-OCT examination using the commercially available equipment Topcon DRI OCT-1 (Topcon Corporation, Tokyo, Japan) without pupil dilation. The macula was scanned using a standard 6×6 mm^2^ protocol, in which 3D acquisition consisted of 256 b-scan slices. The OCT image size was 512×256×992 voxels, with a resolution of 11.72×23.44×2.3 μm^3^. The raw images were exported from the OCT machine in.fds file format for analysis. Some images were included, such as those without eye movements and black bands throughout the scan or other appearances that would impact the subsequent analysis. The OCT image quality indices were provided by the onboard OCT software.

### Image analysis

Eleven surfaces and ten layers were automatically segmented using the 3D graph-based retinal layer segmentation approach [17] applied on the SD-OCT data (Fig 1C), and the original images were flattened to be convenient for quantitative analysis (Fig 1B). The mRNFL, GCIPL, GCC and total retina were analyzed. The segmentation results were reviewed by a retinal specialist, and the images with segmentation errors were excluded.

**Fig 2:**
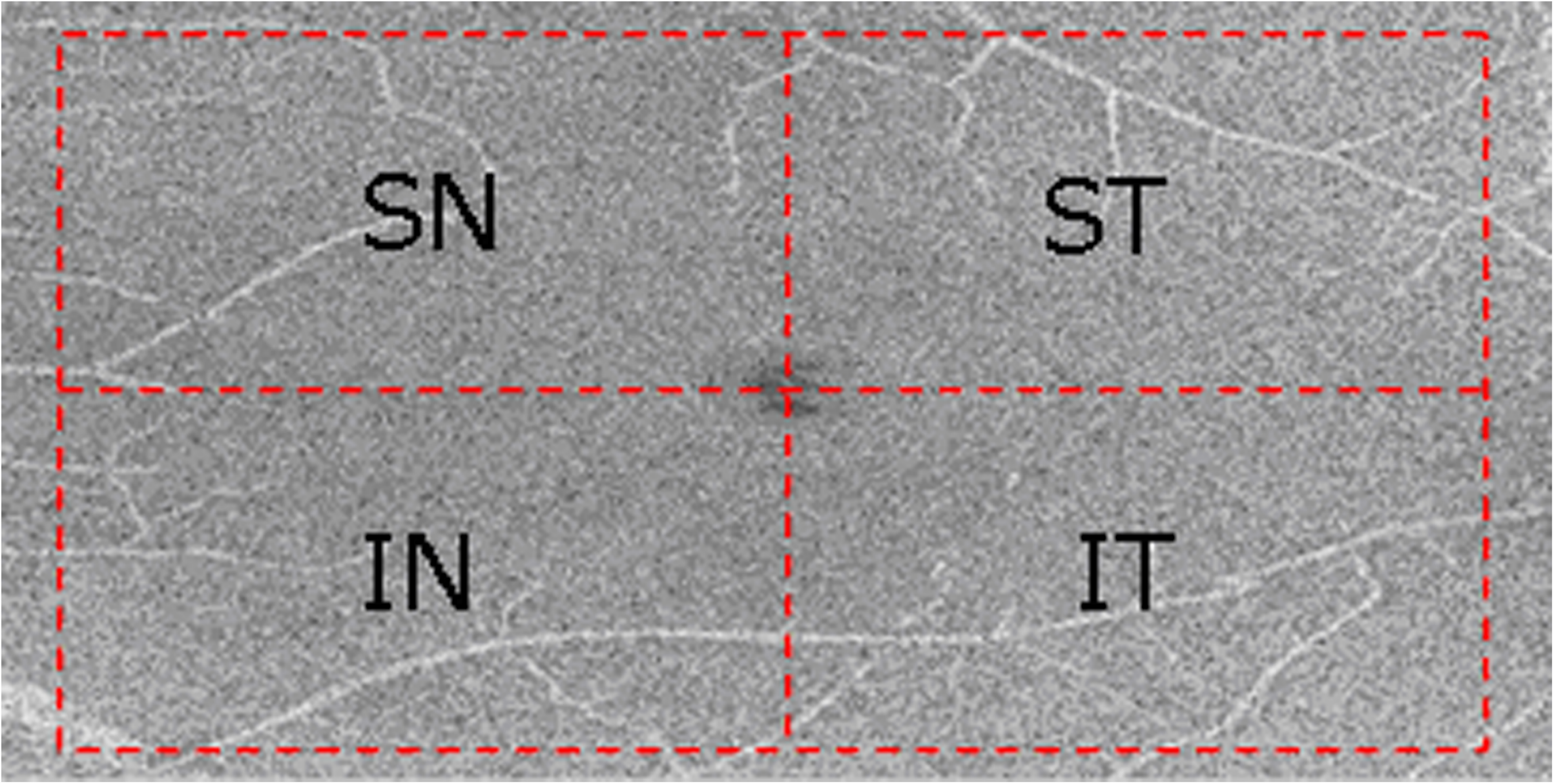
Demarcation of quadrants on the interface between the RNFL and GCL from a left eye.

Due to the scanning focus deviating from the location of the fovea in some OCT data, a square was centered automatically at the fovea by detecting the closest point on the central region of the interface between the RNFL and GCL according to anatomical priors; it had a width of 5.0 mm on the en-face projection available from the 3D SD-OCT volumes (Fig 2), covering the macular area (approximately 5.0 mm in diameter) [18]. To investigate the local characteristics, the square was demarcated into four quadrants [19]: superonasal (SN), inferonasal (IN), superotemporal (ST) and inferotemporal (IT), as shown in Fig 2. Therefore, 5 measurements were made for each layer (i.e., SN, IN, ST, IT and global average).

The thickness, optic intensity ratio and OIAC were calculated for each individual subject according to each layer and quadrant. The thickness of each layer was measured in microns (μm), and was measured by multiplying the resolution (2.3 μm) by the total number of voxels between the top and bottom interfaces of the layer’s z-axis direction. The resulting quadrantal thickness was the average of the thicknesses in each quadrant.

The raw scanned data were interpreted as 16-bit grayscale images resulting in 65,536 levels of gray, with a range from 0 to 65,535. Because raw images were used, intensity was expressed in arbitrary units (AU). The optical intensity ratio of each layer was obtained by normalizing the original intensity with the intensity of the reference layer. Retinal pigment epithelium (RPE) or vitreous is usually used as the reference to normalize the original intensity in optical characteristic measurements of OCT data because RPE has the highest intensity and is assumed to be a uniformly scattering layer, while vitreous has the lowest intensity [10, 13–16]. In addition, outer nuclear layer (ONL) was also found to possess the lowest correlation with image quality in normal eyes, making it the best choice for a baseline medium [12]. In this pituitary adenoma retinal OCT study, we determined the reference layer with the methods presented in Chen *et al* [12]. The resulting quadrantal optical intensity ratio was the average values according to each quadrant.

The OIAC in this paper was defined by modifying the original definition in Vermeer *et al*. [14] as shown in follows:

$$\mu =\frac{\log(\frac{R}{\beta }+1)}{2d}$$ (1)

*R* denotes the ratio of the integrated OCT signal of the layer of interest over the integrated OCT signal of the reference layer, *d* denotes the thickness of the layer of interest, and *β* is a constant. For every A-line in every volumetric scan, values for *R* and *d* were derived and the local attenuation coefficient *μ* were calculated according to Eq (1). The average OIAC in each quadrant of these layers was then calculated. The OIAC was expressed with the metric unit mm^-1^.

### Statistical analysis

The mean and standard deviation of the thickness in each quadrant were calculated by descriptive statistics for the mRNFL, GCIPL and GCC, and total retina for all subjects. The means and standard deviations for the optical intensity ratio and OIAC were also obtained for the RNFL and GCIPL.

The Kolmogorov-Smirnov test was used to study the normality of the data. The data were compared between the patients and the normal controls by Mann-Whitney *U*-test for non-normal variables and unpaired *t*-test for normally distributed variables. Pearson’s correlation was used to evaluate the relationship between the mean intensity of each retinal layer and the image quality score in the determination of the reference layer and to assess the dependency between the optical intensity ratio and the thickness of the mRNFL and GCIPL for patients and controls. Diagnostic performances of the indices were assessed by the receiver operating characteristic (ROC) analysis. The Delong method [20] was employed to evaluate the statistical significance of differences in the area under the ROC curve (AUC) values.

All statistical analyses were performed with the Statistical Package for Social Sciences (SPSS, version 22.0, IBM Corp. Armonk, NY) and MedCalc V.15.2 (Mariakerke, Belgium). A *p-*value less than 0.05 was considered statistically significant. The tables and plots in this paper were drawn by Excel (version 2013, Microsoft, Corp. Redmond, WA), SPSS, and MedCalc.

## Results

A total of 25 left and 13 right eyes of patients between 8–72 years of age and 24 left and 15 right eyes of normal controls between 23–71 years of age were ultimately used in this study. To avoid the correlation between the both eyes of the same subject, only one eye of a subject was included. Pituitary adenoma subjects had no significant age differences compared to the control group (44.66±13.77 years old and 40.79±11.86 years old, p = 0.191), and neither group had significant gender differences with *p*-value of 0.565 (Table 1). The OCT image quality scores of pituitary adenoma subjects had no significant differences compared to those of the control group (p = 0.636) (Table 1).

**Table 1:** Comparison of demographic and image quality information between patients with pituitary adenoma and controls.

	Pituitary adenoma					Control						*P* value
**Age** **(Mean±SD, years)**	44.66±13.77					40.79±11.86						0.191^a^
**Age Range** **(years)**	0–29	30–39	40–49	50–59	≥60		0–29	30–39	40–49	50–59	≥60	
**Gender** **(Male/Female)**	4/1	3/4	6/5	5/5	3/2		7/2	5/4	5/7	0/6	2/1	
	21/17						19/20					0.565^b^
**Total Number**	38						39					
**Image quality**	70.32±6.49						69.31±6.90					0.636^a^

SD, Standard deviation.

^a^ unpaired *t*-test

^b^ χ2 test

Table 2 summarizes the thicknesses measurements in the layers of interest according to quadrant and global average. The mean and local thickness values of each quadrant in the mRNFL, GCC, and total macular retina of the patients with pituitary adenoma were lower than those of controls. The thickness data only partially met normal distribution. Analyzed with appropriate statistical methods, significant differences existed between the groups for thickness measurements in all quadrants of the mRNFL and nasal quadrants of GCC, as well as for the global averages of these layers. The thickness values in the GCIPL of patients were lower compared with controls except those in the IT quadrant, but they were without statistical significance (p = 0.721).

**Table 2:** Comparison of thickness (μm) in the layers of interest and total retina between pituitary adenoma patients and controls according to each quadrant and average of the entire layer.

Macular Parameters		Patient	Control	*P Value*
**mRNFL**	SN	33.70±4.40	37.91±3.79	0.000^a^
	IN	36.20±4.95	41.28±4.97	0.000^b^
	ST	21.65±2.49	23.17±2.17	0.005^b^
	IT	23.73±2.94	25.03±2.16	0.039^b^
	Average	28.84±3.22	31.82±2.62	0.000^a^
**GCIPL**	SN	65.25±7.37	67.53±4.94	0.349^b^
	IN	61.92±7.14	64.73±4.72	0.157^b^
	ST	65.93±5.77	66.07±4.32	0.907^a^
	IT	66.00±5.51	65.90±4.37	0.721^b^
	Average	64.76±6.06	66.06±4.39	0.283^a^
**GCC**	SN	98.94±10.83	105.43±6.13	0.004^b^
	IN	98.10±10.31	106.00±6.60	0.000^b^
	ST	87.57±7.49	89.23±5.35	0.266^a^
	IT	89.72±7.54	90.92±5.32	0.420^a^
	Average	93.59±8.54	97.88±5.27	0.015^b^
**Total Retina**	SN	277.58±14.54	282.25±10.01	0.157^b^
	IN	268.68±14.68	275.28±10.13	0.061^b^
	ST	259.55±14.72	261.81±9.58	0.548^b^
	IT	257.44±13.36	258.57±9.90	0.951^b^
	Average	265.85±13.78	269.48±9.43	0.333^b^

mRNFL, macular retinal nerve fiber layer; GCIPL, ganglion cell and inner plexiform layer; GCC, ganglion cell complex; SN, superonasal; IN, inferonasal; ST, superotemporal; IT, inferotemporal.

^a^ unpaired *t*-test

^b^ Mann-Whitney *U* test

Mean ± Standard deviation

Table 3 shows the process of the reference layer identification. Because of the lower variances after adjustment of correlation, the vitreous, ONL, and RPE (with values of 0.0096, 0.0129, and 0.0130, respectively) were verified to be slightly correlated with the image quality index. Using the standard deviation of intensity and correlation coefficient among these three layers, RPE was determined to be the reference layer because it performed the best. In addition, this layer would not be influenced by the chiasmal lesion induced by the pituitary adenoma or pituitary adenoma *per se*.

**Table 3:** Mean and standard deviation of optical intensities in different layers.

Retinal Layer	Mean Intensity	SD	Variance	*r*^2^	Adjusted Variance	Adjusted SD	Adjusted Coefficient of Variation
**Vitreous**	14611.2	148.7	22108.4	10.1%	19875.4	141.0	0.0096
**RNFL**	29603.9	944.5	892047.7	35.0%	579831.0	761.5	0.0257
**GCIPL**	27627.8	1190.7	1417743.5	69.8%	428158.5	654.3	0.0237
**INL**	24265.7	1208.8	1461076.8	83.5%	241077.7	491.0	0.0202
**OPL**	25971.5	1256.1	1577671.3	84.6%	242961.4	492.9	0.0190
**ONL**	22402.5	1144.1	1309014.5	93.6%	83776.9	289.4	0.0129
**ELM**	31042.8	1328.8	1765682.2	75.4%	434357.8	659.1	0.0212
**ISOS**	35128.8	1548.6	2398065.9	51.9%	1153469.7	1074.0	0.0306
**OS**	34822.7	1350.7	1824428.4	51.3%	888496.6	942.6	0.0271
**RPE**	37704.2	718.2	515772.1	53.2%	241381.3	491.3	0.0130
**ALL areas**	18169.6	345.5	119374.4	9.9%	107556.3	328.0	0.0180

RNFL, retinal nerve fiber layer; GCIPL, ganglion cells and inner plexiform layer; INL, inner nuclear layer; OPL, outer plexiform layer; ONL, outer nuclear layer; ELM, external limiting membrane; ISOS, photoreceptor inner/outer segment layers; OS, outer photoreceptor segment layer; RPE, retinal pigment epithelium; SD, standard deviation; *r*, coefficient of determination with image quality.

Table 4 presents the mean and standard deviation of the optical intensity ratio in the mRNFL and GCIPL according to quadrant and global average. The mean values of the optical intensity ratios were decreased in most quadrants of the retinal layers in the patients compared with controls, except in the ST quadrant of the mRNFL. However, none of the differences reached statistical significance.

**Table 4:** Comparison of optical intensity ratio in the mRNFL and GCIPL between pituitary adenoma patients and controls.

**Macular Parameters**		**Patient**	**Control**	***P* Value**
**mRNFL**	**SN**	0.710±0.026	0.718±0.029	0.176
	**IN**	0.724±0.027	0.731±0.027	0.284
	**ST**	0.672±0.029	0.667±0.028	0.465
	**IT**	0.683±0.028	0.685±0.027	0.774
	**Average**	0.697±0.024	0.700±0.025	0.600
**GCIPL**	**SN**	0.646±0.028	0.652±0.028	0.378
	**IN**	0.660±0.030	0.662±0.027	0.750
	**ST**	0.636±0.029	0.645±0.026	0.184
	**IT**	0.643±0.027	0.654±0.027	0.078
	**Average**	0.646±0.027	0.653±0.025	0.263

mRNFL, macular retinal nerve fiber layer; GCIPL, ganglion cells and inner plexiform layer; SN, superonasal; IN, inferonasal; ST, superotemporal; IT, inferotemporal.

unpaired *t*-test

Mean±Standard deviation

The relationships between the optical intensity ratio and the thickness of the mRNFL and GCIPL are shown in Table 5 for patients and controls. The optical intensity ratios presented significant positive correlations with the thickness measurements in the SN quadrants of the mRNFL for both groups (Pearson’s coefficient = 0.371, p = 0.022 for patients and Pearson’s coefficient = 0.357, p = 0.026 for controls) and the IN quadrant of the mRNFL in controls (Pearson’s coefficient = 0.411, p = 0.009). There were significant negative correlations between these two measurements in almost all quadrants of the GCIPL in the patients group except for the ST quadrant (p = 0.093). We also noted that positive correlations existed in the ST and IT quadrants and global average of the mRNFL in both groups, as well as in almost all quadrants of the GCIPL from the controls group except the IN quadrant, but they were without statistical significance.

**Table 5:** Correlation analysis between the optical intensity ratio and the thickness of the mRNFL and GCIPL for pituitary adenoma patients and controls.

Macular Parameters		Patient		Control	
		coefficient	*P* value	coefficient	*P* value
**mRNFL**	SN	0.371	0.022	0.357	0.026
	IN	0.318	0.052	0.411	0.009
	ST	0.321	0.050	0.263	0.106
	IT	0.273	0.097	0.262	0.107
	Average	0.238	0.151	0.258	0.112
**GCIPL**	SN	-0.400	0.013	0.130	0.429
	IN	-0.382	0.018	-0.115	0.486
	ST	-0.277	0.093	0.055	0.739
	IT	-0.327	0.045	0.053	0.746
	Average	-0.351	0.031	0.038	0.819

mRNFL, macular retinal nerve fiber layer; GCIPL, ganglion cells and inner plexiform layer; SN, superonasal; IN, inferonasal; ST, superotemporal; IT, inferotemporal.

Table 6 demonstrates the mean and standard deviation of the OIAC in the mRNFL and GCIPL for each quadrant. In this paper, *β* is 2.3, and it was obtained by optimal fitting according to the method described by Vermeer *et al* [14]. The OIAC measurements in all quadrants were higher for patients than for controls, except in the IT quadrant of the GCIPL (p = 0.354). Statistically significant differences existed between the groups in the SN quadrant (p = 0.014), ST quadrant (p = 0.021), and average value of the mRNFL (p = 0.042).

**Table 6:** Comparison of OIACs (mm^-1^) in the mRNFL and GCIPL between pituitary adenoma patients and controls.

Macular Parameters		Patient	Control	*P* Value
**mRNFL**	**SN**	7.38±0.38	7.15±0.41	0.014^a^
	**IN**	7.29±0.40	7.14±0.39	0.172^b^
	**ST**	8.02±0.55	7.76±0.41	0.021^a^
	**IT**	7.84±0.48	7.74±0.47	0.596^b^
	**Average**	7.63±0.42	7.45±0.35	0.042^a^
**GCIPL**	**SN**	5.59±0.30	5.51±0.30	0.247^a^
	**IN**	5.73±0.34	5.69±0.28	0.561^a^
	**ST**	5.62±0.33	5.61±0.23	0.938^a^
	**IT**	5.59±0.33	5.66±0.27	0.354^a^
	**Average**	5.63±0.31	5.62±0.24	0.798^a^

mRNFL, macular retinal nerve fiber layer; GCIPL, ganglion cells and inner plexiform layer; SN, superonasal; IN, inferonasal; ST, superotemporal; IT, inferotemporal.

^a^ unpaired *t*-test

^b^ Mann-Whitney *U* test

Mean±Standard deviation

**Fig 3:**
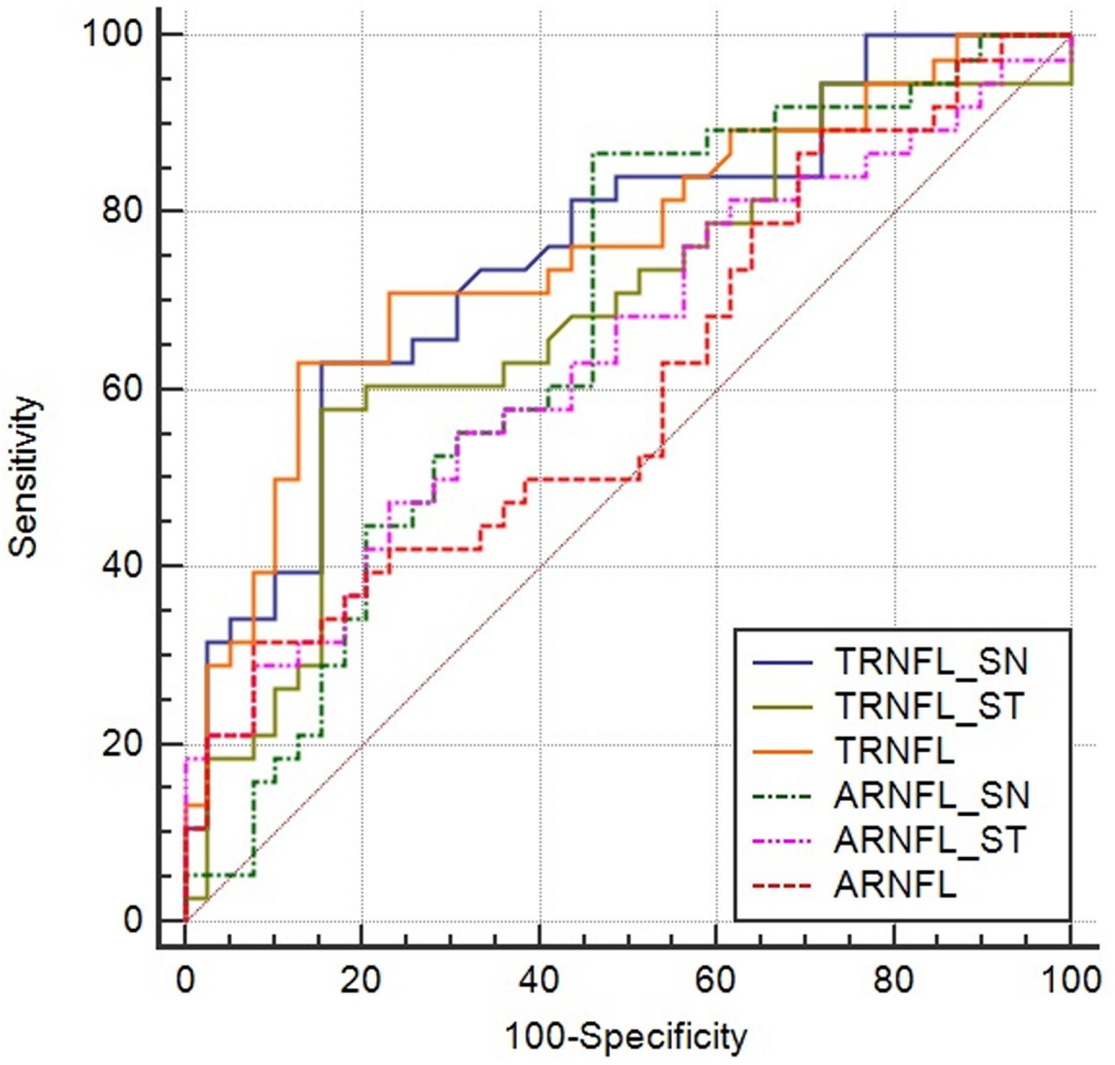
Receiver operating characteristic curves (ROC) of the thickness and OIAC measurements in the SN quadrant, ST quadrant, and global average of mRNFL (expressed as TRNFL_SN, TRNFL_ST, TRNFL, and ARNFL_SN, ARNFL_ST, ARNFL, respectively).

Table 7 shows the results, which present statistically significant differences, of comparisons of the area under the ROC curves (AUC) between pituitary adenoma patients and controls. Thickness in the IN quadrants of the mRNFL presented the greatest AUC value (0.764), followed by the mRNFL thickness in global average (AUC value = 0.759), SN quadrant (AUC value = 0.758), and IN quadrant of the GCC (AUC value = 0.743) with a *p* value less than 0.0001. The mRNFL thickness in the SN and ST quadrants and global average had better diagnostic accuracy than the OIAC of those areas (Table 7). The AUC of the global average mRNFL thickness was significantly higher than that of the global average OIAC in the mRNFL for discriminating pituitary adenoma patients from normal controls (p = 0.0265) (Fig 3 and Table 8).

**Table 7:** Comparison of the area under the receiver operating characteristic curves (AUC) between pituitary adenoma patients and controls.

Macular Parameters			Area under curve(SE)	95% CI	z statistic	*P* value
**Thickness**	**mRNFL**	**SN**	0.758(0.0551)	0.647–0.848	4.674	<0.0001
		**IN**	0.764(0.0546)	0.653–0.853	4.837	<0.0001
		**ST**	0.687(0.0619)	0.571–0.788	3.026	0.0025
		**IT**	0.637(0.0651)	0.519–0.744	2.105	0.0353
		**Average**	0.759(0.0556)	0.649–0.850	4.666	<0.0001
	**GCC**	**SN**	0.691(0.0604)	0.575–0.791	3.159	0.0016
		**IN**	0.743(0.0579)	0.631–0.836	4.198	<0.0001
		**Average**	0.661(0.0632)	0.544–0.765	2.552	0.0107
**OIAC**	**mRNFL**	**SN**	0.670(0.0627)	0.554–0.773	2.712	0.0067
		**ST**	0.642(0.0635)	0.525–0.748	2.243	0.0249
		**Average**	0.607(0.0651)	0.489–0.717	1.649	0.0991

mRNFL, macular retinal nerve fiber layer; GCC, ganglion cell complex; SN, superonasal; IN, inferonasal; ST, superotemporal; IT, inferotemporal; OIAC, optical intensity attenuation coefficient; SE, standard error, CI, confidence interval.

**Table 8:** Pairwise comparison of the area under the receiver operating characteristic curves (AUC) between the thickness and OIAC measurements in the SN quadrant, ST quadrant, and global average of mRNFL.

	Difference between AUC (SE)	95% CI	z statistic	P value
**SN**	0.0877(0.0704)	-0.0502–0.226	1.246	0.2126
**ST**	0.0449(0.0830)	-0.118–0.207	0.541	0.5886
**Average**	0.152(0.0686)	0.0177–0.287	2.219	0.0265

SN, superonasal; ST, superotemporal; SE, standard error, CI, confidence interval.

## Discussion

In this study, we demonstrated the quantitative analysis for 3D SD-OCT retinal data of patients with pituitary adenoma and healthy controls using three parameters: thickness, optical intensity ratio, and OIAC. To the best of our knowledge, this study is the first to analyze the optical reflective characteristics of the retinal OCT data of patients with pituitary adenoma. We discovered the effectiveness of mRNFL thickness in distinguishing patients with pituitary adenoma from normal controls and the limited diagnostic ability of the optical characteristic index.

The macula contains a large proportion of retinal ganglion cell (RGC) neurons (approximately 34% of total macular volume) [21] and glial cells. Measurements of macular thickness and volume reflect the integrity of retinal neurons and quantify neuronal loss [22]. We studied the retinal layers that have relationship with the RGCs: the RNFL consists of ganglion cells axons, the GCL consists of ganglion cell bodies, and the IPL consists of ganglion cell dendrites [23]. For the similar intensity presented in the OCT reflectivity profile, the GCL and IPL are usually analyzed together. When the optic chiasm is directly compressed or its blood supply system is interfered with by the pituitary adenoma, axonal injury and dysfunction and/or apoptosis to the RGCs may occur [2, 24–27], resulting in the thinning of the RNFL and GCIPL. The nasal hemiretina is predominantly affected by its association with the crossed nerve fibers in the optic chiasm. With the variation of lesion location and compressed position in the optic chiasm, as well as aggravation of damage to the optic chiasm, the temporal hemiretina is also thinned in response to the injured fibers. From the results of this analysis, we found that the thicknesses in all quadrants of the mRNFL were significantly decreased in patients compared with healthy subjects, particularly in the SN and IN quadrants (Table 2). The nasal quadrants of the GCC presented statistically significant thickness differences in patients compared with controls, further affecting the loss of RGCs (Table 2).

Morphological structural changes in the macular area of the pituitary adenoma retina were found by the thickness measurements. To reveal the pathological structural variations, we performed two optic quantitative analyses, including the optical intensity ratio and OIAC. We found that the optical intensity ratio measurements were lower in almost all the layers of interest patients than those in normal controls, although without significant differences (Table 4). This phenomenon of decreasing optical intensity in the RNFL of pituitary adenoma patients was coincident to the optical reflective situation of the glaucomatous RNFL, which also suffers impairments to the nerve fibers and thinning of the RNFL [15, 16]. The variations in the optical intensity of the retinal layer were proven to exhibit functional changes in the RGCs [28]. When RGC dysfunction emerges but is not severe enough to cause the RGCs to die, the density of nerve fibers decreases and the RNFL has lower reflectivity presenting lower optical intensity than that in normal RGCs; sometimes there may be no changes in the thickness. This may explain the non-significance of the intensity difference between the patients and controls (Table 4) and the non-consistent significant positive correlations which were found in the analysis between the optical intensity and the thickness for those groups (Table 5). It is noted that the optical intensity ratio in the GCIPL slightly diminished and significantly decreased along with the increase of thickness in almost all quadrants of the GCIPL in the retinas of the patients group (Tables 4 and 5). The modulation of OCT reflectivity properties is affected not only by the retinal layer’s structure, including cell number and distribution, but also by the chemical gradient [29]. Variation in the organelles during the process of RGC apoptosis or necrosis could be an important reason for the phenomenon [30, 31], but the exact cellular and molecular mechanism for changes in optical intensity of the GCIPL in retinas with pituitary adenoma remains unknown and deserves further investigation.

According to the results of the quantitative analysis, the OIAC values are higher in most quadrants of patients than controls except the inferotemporal GCIPL (Table 6). This is contrary to some conclusions reached in other studies of damaged RGCs [14, 16]. However, the finding is reasonable in this paper because the decreases of layer thickness were more remarkable than those of the optical intensity ratio in the calculations of OIAC. The OIAC was proposed to quantify the optical scattering properties from OCT data [16], manifesting both morphologic and optical characteristic information. It could be more comprehensive than either thickness or optical intensity ratio. However, the OIAC was proven to have worse diagnostic capability than thickness in the comparison of AUC values between pituitary adenoma patients and controls (Tables 7 and 8). The thickness and optical intensity ratio evaluate the state of the retina from another perspective and are more intuitive for expressing the variations induced by diseases in the retina. In particular, the nasal hemiretinal thicknesses showed excellent performances in distinguishing the patients suffering pituitary adenoma from controls (Table 7), which conformed to the pathology of the optic chiasm lesion. It is possible that the evaluation using the combination of morphological and optical characteristic information provided by SD-OCT data should be formed differently.

The correlations between the morphological and optical characteristic parameters and functional visual loss could be analyzed with clinical relevant information, such as visual field defect measurements. Future studies will further explore the relationships among variations in retinal thickness, optical reflectivity and visual function.

In conclusion, based on the quantitative investigations, the measurements of retinal thickness were more sensitive than optical characteristics in this study of pituitary adenoma. The thicknesses of mRNFL and nasal GCC were significantly decreased in the retina of patients with pituitary adenoma compared with controls, while the optical intensity ratio and OIAC had partial statistical significance. In patients, optical intensity ratios were increased in mRNFL but decreased in GCIPL along with an increase of retinal thicknesses. Comparison of the AUC demonstrated that the thickness of the mRNFL could be used as an aided diagnostic index in distinguishing pituitary adenoma patients from normal people, and the optical characteristic parameters had limited diagnostic abilities. The development of a more comprehensive and reasonable quantitative index that uses the optical characteristics is encouraged for future studies.
